# Exosomal hsa-miR-299-3p from endothelium mediates high phosphorus-induced vascular calcification in mice model of CKD via phosphorylated JAK2/STAT5 pathway

**DOI:** 10.3389/fphar.2026.1752954

**Published:** 2026-04-09

**Authors:** Si-Jie Chen, Tao-Tao Tang, Li-Jun Xie, Bo Wang, Yan Di, Min Wu, Zuo-Lin Li, Yi Wen, Lin-Li Lv, Ri-Ning Tang, Bin Wang, Bi-Cheng Liu

**Affiliations:** Institute of Nephrology, Zhongda Hospital, School of Medicine, Southeast University, Nanjing, Jiangsu, China

**Keywords:** CKD, exosomes, hsa-miRNA-299-3p, MARCH3, vascular calcification

## Abstract

**Introduction:**

Vascular calcification (VC) is a prevalent and life-threatening complication of chronic kidney disease (CKD), yet the mechanisms by which hyperphosphatemia drives VC remain incompletely understood. This study investigates the role of endothelial cells (ECs)-derived exosomal microRNAs in mediating osteogenic differentiation of vascular smooth muscle cells (VSMCs) under high phosphate (HP) conditions.

**Methods:**

A CKD-VC mouse model was established using a HP and high-adenine diet. Exosomes (Exos) were isolated from ECs cultured under normal or HP conditions. The effects of Exos on calcification of VSMCs were evaluated using *in vitro* co-culture systems and *in vivo* administration. miRNA sequencing, dual-luciferase reporter assays, and loss/gain of function experiments were performed to identify key exosomal miRNAs and their downstream targets. Western blotting, qRT-PCR, and histological analyses were used to assess molecular and pathological changes.

**Results:**

HP-stimulated ECs released Exos (HP-Exos) that were internalized by VSMCs and significantly promoted VC in both *in vitro* and *in vivo* models. miRNA sequencing identified miR-299-3p as significantly upregulated in HP-Exos. Functional studies demonstrated that exosomal miR-299-3p directly targeted membrane-associated RING-CH3 (MARCH3), leading to activation of the p-JAK2/STAT5 signaling pathway. This cascade subsequently upregulated osteogenic markers and downregulated contractile marker, thereby promoting osteogenic differentiation of VSMCs. Knockdown of miR-299-3p *in vivo* attenuated VC in CKD mice.

**Discussion:**

These findings reveal a previously unrecognized mechanism by which HP drives CKD-VC through ECs-derived exosomal miR-299-3p. The miR-299-3p/MARCH3/p-JAK2/STAT5 signaling axis represents a critical regulatory pathway in VC pathogenesis and offers a potential therapeutic target for this life-threatening complication of CKD.

## Introduction

Vascular calcification (VC) is one of the most common complications of chronic kidney disease (CKD), standing out as a potent predictor of cardiovascular mortality (GBD, 2020; Shlipak et al., 2021; Kovesdy, 2011; Xie et al., 2024). It was shown that patients with CKD exhibiting a striking three fold higher incidence of VC compared to age-matched controls (Jankowski et al., 2021). This pathological process involves the aberrant deposition of hydroxyapatite crystals within the vascular wall, particularly in the tunica media, where it triggers a dramatic phenotypic transformation of vascular smooth muscle cells (VSMCs) (Onnis et al., 2024; Li et al., 2024). Our previous work identified that miR-29a/Notch1 and miR-214-3p/CXCL8 as contributors to this process (Wang et al., 2021; Wang L. T. et al., 2022), while the fundamental triggers initiating this phenotypic switch remain enigmatic.

With the progression of CKD, hyperphosphatemia emerging as a key instigator of VC. Studies suggested that elevated serum phosphate levels directly activated osteogenic programming in VSMCs, accelerating medial calcification (Chen et al., 2024). Importantly, severity of VC independently predicts cardiovascular outcomes in CKD patients, highlighting the urgent need to unravel its molecular mechanisms. As the vascular sentinels, endothelial cells (ECs) occupy a privileged position to sense and respond to phosphate fluctuations. Mounting evidence suggests that high phosphate (HP) activated ECs regulate VC progression through paracrine communication with neighboring VSMCs (Judith and Sluimer, 2024; Zhang et al., 2023; Moncla et al., 2023; Niu et al., 2024). However, the nature of these critical ECs-derived signals remains poorly characterized.

Recent breakthroughs have implicated exosomes (Exos), rich in bioactive cargo, as key mediators of intercellular communication in vascular pathology (Tang et al., 2020). ECs-derived Exos under HP conditions have been tentatively linked to VC progression (Shan et al., 2024; Chiva-Blanch and Davidson, 2022; Guo et al., 2022), with specific effectors like miR-670-3p/IGF-1 and HMGB1 shown to promote dysfunction of VSMCs (Lin et al., 2022; Boyer et al., 2020). Yet, a systematic understanding of how HP-stimulated Exos (HP-Exos) drive osteogenic transdifferentiation in CKD remains lacking.

Here, we unveil a novel signaling axis centered on exosomal hsa-miR-299-3p that bridges HP stress to VC development in CKD. Through integrative approaches, we demonstrate that HP-Exos serve as efficient vehicles for miR-299-3p delivery to VSMCs, and miR-299-3p could directly target membrane-associated RING-CH3 (MARCH3) by activating phosphorylated(p)-JAK2/STAT5 signaling, to stimulate the formation of VC. Our findings establish a previously unrecognized miR-299-3p/MARCH3/p-JAK2/STAT5 pathway as the central regulatory mechanism in CKD-associated VC (CKD-VC), which offer a potential therapeutic target for this life threatening complication.

## Materials and methods

### Cell culture

Primary human umbilical vein endothelial cells (HUVECs) were isolated from umbilical cords obtained from Zhongda Hospital (Ethical Approval No. 2023ZDSYLL118-P01) and cultured in endothelial cell medium (ECM, ScienCell Research Laboratories) supplemented with 10% fetal bovine serum (FBS, Gibco). Human aortic vascular smooth muscle cells (VSMCs) were procured from the American Type Culture Collection (ATCC) and maintained in Dulbecco’s Modified Eagle Medium/Nutrient Mixture F-12 (DMEM/F12, Gibco) containing 10% FBS. All cells were incubated at 37 °C in a humidified atmosphere with 5% CO2. Upon reaching 70% confluence, cells were treated with HP medium containing inorganic phosphate (3 mmol/L) for 7 days to induce calcification. All *in vitro* experiments were conducted with a minimum of three independent replicates to ensure statistical reliability and biological reproducibility.

#### Transwell co-culture system

A Transwell co-culture system was employed to investigate cell-cell interactions. HUVECs, pre-treated with the Exos inhibitor GW4869 (Sigma-Aldrich) for 24 h, were seeded into the upper chamber of Transwell inserts (0.4 µm pore size, Corning, United States), while VSMCs were cultured in the lower chamber. The co-culture system was maintained under HP conditions for 7 days.

## Cell transfection

To modulate gene expression, hsa-miR-299-3p mimics, inhibitors, and three short hairpin RNA (shRNA) sequences targeting MARCH3 were designed and synthesized by GenePharma (China). Transfection was performed using Lipofectamine 3,000 (Invitrogen, United States), with hsa-miR-299-3p mimic, inhibitor, or their respective negative controls (100 nM) introduced into the cells for 48 h. MARCH3 knockdown in VSMCs was achieved using shMARCH3.

### Isolation and characterization of exos

Exos were isolated from the conditioned medium of ECs exposed to either normal or HP conditions for 48 h. The supernatant was subjected to sequential centrifugation steps: 2,000 × g for 20 min to remove cellular debris, 15,000 × g for 30 min to eliminate larger vesicles, and ultracentrifugation at 100,000 × g for 2 h (Type 45Ti ultracentrifuge, Beckman, Germany) to pellet Exos. The isolated Exos were characterized for morphology using transmission electron microscopy (TEM) (JEM1200EX, JEOL, Japan), size distribution via nanoflow cytometry (NFC) (NanoFCM, China), and exosomal marker expression by Western blot.

### Uptake of exos

For *in vitro* uptake studies, Exos were labeled with the near-infrared fluorescent dye DIR (Sigma-Aldrich). Briefly, DIR (1 mM) stock solution was prepared in ethanol, and the dye (2 µL) was incubated with the Exos suspension for 25 min at 37 °C with gentle agitation. Labeled Exos were washed with PBS and pelleted by ultracentrifugation at 100,000 × g for 1 h at 4 °C. VSMCs were incubated with DIR-labeled Exos (10 μg/mL) for 12 h, fixed with 4% paraformaldehyde, and counterstained with DAPI (1 μg/mL, Sigma-Aldrich) for nuclear visualization. Cellular uptake was assessed using a confocal laser scanning microscope (FV3000, Olympus).

For *in vivo* tracking, DIR-labeled Exos (100 µg in 100 µL PBS) or PBS (control) were administered intravenously to CKD mice. At 24 h post-injection, fluorescence signals were captured using the IVIS Spectrum Imaging System (PerkinElmer) with near-infrared filters (excitation/emission: 748/780 nm). Mice were anesthetized with 2% isoflurane during imaging. Fluorescence intensity was quantified using Living Image software (PerkinElmer). Subsequently, aortas were harvested for *ex vivo* imaging.

## Animal experiments

Animal experiments were approved by the Ethical Committee of Southeast University (Approval No: 20220224039). Male C57BL/6 mice (8 weeks old, 20–24 g) were fed under controlled conditions (12-h light/dark cycle, 23 °C–25 °C, 50%–60% humidity, noise <60 dB). The animal study used 5 subjects per group, following standard practice to ensure validity while minimizing animal use. This sample size accounts for biological variability and detects meaningful differences without formal power calculations, aligning with ethical and scientific guidelines. The control group received a standard diet for 16 weeks, while the CKD group was fed a standard diet for 2 weeks, followed by 0.2% adenine for 4 weeks, and a combined diet of 1.8% phosphate and 0.2% adenine for the remaining 10 weeks. CKD mice were intravenously injected with normal phosphate-derived Exos (NP-Exos) or HP-Exos (100 µg per dose, twice weekly) until the end of the study.

For miRNA inhibition, an shRNA targeting miR-299-3p was cloned into the adeno-associated virus (AAV)-U6-shRNA vector (GenePharma) and packaged into AAV9 for tail vein injection. Mice were injected intravenously via the tail vein, then fed the same diet as CKD group. After 16 weeks, mice were anesthetized with 2% isoflurane in oxygen via a nasal cone connected to an isoflurane anesthesia machine (E-Z Anesthesia, Euthanex Corp, PA, United States). Mice were anesthetized for 4 min and maintained anesthesia for tissue collection. Then, mice were euthanized using a cervical dislocation method.

### Calcium content and alkaline phosphatase (ALP) activity detection

Calcium levels in cell lysates or tissues were quantified using a calcium assay kit. Samples were centrifuged to remove particulates and diluted with Calcium Assay Buffer to ensure readings fell within the standard curve range. Standards and samples (50 µL each) were loaded into a microplate, followed by the addition of Calcium Chromogenic Reagent (150 µL). Absorbance was measured at 575 nm within 10 min.

ALP activity was determined using a commercial kit (Beyotime). Samples (30 µL) were mixed with assay buffer (50 µL) and substrate solution (50 µL), followed by color developer (150 µL). Absorbance was read at 520 nm within 30 min.

#### Quantitative real-time PCR (qRT-PCR)

After 7 days, cells were collected. Total RNA was extracted using TRIzol reagent (Invitrogen) and quantified with a NanoDrop 2000 spectrophotometer (Thermo Fisher Scientific). cDNA synthesis was performed using RT SuperMix (Vazyme) and a miRNA-specific cDNA kit (Vazyme). Gene expression was analyzed on a 7,300 qRT-PCR PCR System (Applied Biosystems) with primers listed in Supplementary Tables S1, S2. Relative mRNA levels were calculated using the 2^−ΔΔCT^ method.

### MiRNA sequencing

Small RNA libraries were prepared and sequenced on an Illumina NovaSeq 6,000 (Echobiotech, China). Raw reads were demultiplexed using bcl2fastq (Illumina), and adapter sequences were trimmed with Cutadapt. Clean reads were aligned to the GRCh38 genome, and miRNA expression was quantified with miRDeep2. Differentially expressed miRNAs were defined as those with a fold change ≥2 and a false discovery rate (FDR) < 0.05.

### Dual-luciferase reporter assay

The 3′untranslated region (3′UTR) of MARCH3 was cloned into the pGL3 luciferase vector (GenePharma). A mutant 3′UTR (pGL3-MARCH3-3′UTR-MUT) was generated using a mutagenesis kit (Agilent Technologies). HEK293T cells were transfected with reporter plasmids for 48 h, and luciferase activity was measured using a Dual-Luciferase Reporter Assay System (GenePharma).

### Western blot

After 7 days, cells were collected. Proteins were extracted using RIPA lysis buffer (Beyotime, China) supplemented with complete protease inhibitor cocktail on ice for 30 min, followed by centrifugation at 12,000 × g for 15 min at 4 °C to collect supernatants. Protein concentrations were determined using a BCA assay kit according to the manufacturer’s protocol. Equal amounts of protein were separated by 10% SDS-PAGE electrophoresis at 80 V for 30 min followed by 120 V for 1–1.5 h, then transferred to PVDF membranes (Millipore, United States) at 300 mA for 1.5 h in ice-cold transfer buffer. After blocking with 5% non-fat milk in TBST for 1 h at room temperature, membranes were incubated with primary antibodies (Supplementary Table S3) overnight at 4 °C with gentle shaking. Following three washes with TBST (10 min each), membranes were incubated with appropriate HRP-conjugated secondary antibodies for 1 h at room temperature. Protein bands were visualized using the ECL Plus detection system (GE Healthcare, United States) and quantified by densitometry analysis with ImageJ software (NIH, United States), with β-actin or GAPDH serving as loading controls.

### Vascular histology

4% paraformaldehyde was applied to fix aortic tissues from mice. They were cut into 5 µm sections of thickness. Sequential xylene and ethanol treatments were deparaffinized, followed by rinsing with distilled water. The sections were incubated with silver nitrate solution for 25 min, then sodium thiosulfate solution for 2 min to perform Vonkossa staining.

#### Kidney histology

4% paraformaldehyde was applied to fix kidney tissues from mice for 24 h and subsequently dehydrated through a series of ethanol solutions with increasing concentrations. The dehydrated tissues were cleared in xylene and embedded in paraffin blocks. They were cut into thickness slides (4 µm). Then we used Masson’s trichrome and hematoxylin and eosin (H&E) for staining.

#### Immunohistochemistry (IHC)

Prior to being sliced into thick sections (4 µm), aortic tissues were preserved in 4% paraformaldehyde, underwent dehydration via a series of ethanol treatments, and were subsequently embedded in paraffin. These paraffin-embedded sections were deparaffinized, rehydrated, underwent antigen retrieval and bocked with 5% bovine serum albumin (BSA). They were incubated with anti-MARCH3 primary antibodies (Affinity Biosciences, DF4019), followed by exposure to HRP-conjugated secondary antibodies.

### Alizarin Red S staining

VSMCs were fixed with 4% paraformaldehyde for 20 min, washed three times with PBS, and then stained with Alizarin Red solution at room temperature for 15 min in dark. After thorough rinsing with ultrapure water, calcium nodules were observed under a microscope.

### X-ray diffraction analysis

Calcified aortic tissues were ground into powder and analyzed using an X-ray diffractometer (Bruker D8 Advance, Beijing Zhongke Baice Technology, China). The scanning range and step angle were set, and diffraction peaks were analyzed to determine crystal structure information using Bragg’s law.

### Statistical analysis

All statistical analyzes were performed using GraphPad Prism 9.0 (GraphPad Software Inc., San Diego, CA, United States). Data were presented as mean ± standard deviation (SD). Two-tailed unpaired Student’s t-test was applied for analyzing comparisons between two groups. One-way ANOVA followed by Bonferroni correction was applied for analyzing comparisons among three or more groups. Statistical significance was defined as p < 0.05. P value style: <0.05 (*), <0.01 (**), <0.001 (***), <0.0001 (****), ns: no significance.

## Results

### Exos derived from HP stimulated ECs could be uptaken by CKD mice

**FIGURE 1:**
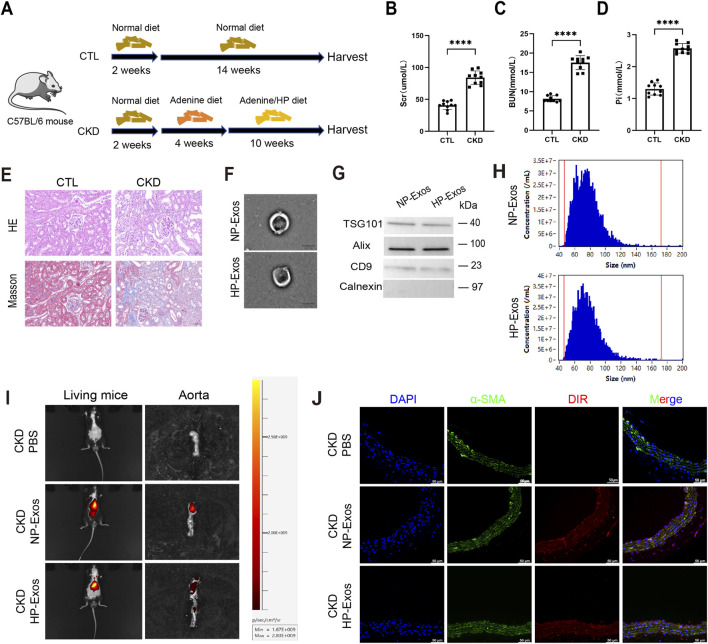
Exos derived from HP exposed ECs could be uptaken by CKD mice. **(A)** CKD-VC model by subjecting mice to a HP, high-adenine diet for 16 weeks (n = 10 per group). **(B)** Serum creatinine (Scr) content of mice was determined. **(C)** Serum BUN content of mice was determined. **(D)** Serum Pi content of mice was determined. **(E)** HE and Masson pathological staining of kidney (n = 10 per group, scale bar = 50 μm). **(F-H)** Exos were isolated from the supernatants of ECs cultured with or without HP (3 mmol/L) through ultracentrifugation (n = 3 per group). **(F)** Structure of Exos was detected by transmission electron microscopy (scale bar = 100 nm). **(G)** The protein levels of CD9, Alix, TSG101 and Calnexin of Exos in the control group or HP group were indicated by Western blot. **(H)** Size distribution of Exos was detected by nanoflow analysis. **(I,J)** DIR labeled Exos were injected through the tail vein of mice (n = 5 per group). **(I)** After 24 h of injection, fluorescence signals were detected in the living mice (left panel). The fluorescence signal of arota was detected after the mice were killed (right panel). **(J)** The uptake of ECs-derived Exos was analyzed in aorta sections. Blue fluorescence (DAPI) marks the nucleus, green fluorescence marks the α-SMA of VSMCs and red fluorescence marks the Exos (scale bar = 50 μm). Data represented mean ± SD, unpaired two-tailed Student’s t-test. ****p < 0.0001.

We established a mouse model of CKD by feeding a HP and high-adenine diet for 16 weeks (Figure 1A). Compared to the control group, this dietary regimen significantly increased serum levels of creatinine (Scr), blood urea nitrogen (BUN), and inorganic phosphate (Pi) in the CKD group (Figures 1B–D). Histological analysis demonstrated marked renal injury, characterized by inflammatory cell infiltration and interstitial fibrosis (Figure 1E).

Exos were isolated from the supernatants of ECs cultured with or without HP conditions. TEM revealed the characteristic bilayer membrane structure of Exos (Figure 1F). Both HP-treated and nonHP-treated groups exhibited positive expression of exosomal markers (TSG101, CD9, and Alix), while calnexin was undetectable (Figure 1G). Nanoparticle tracking analysis showed a typical unimodal size distribution, with diameters ranging from 50 to 150 nm (Figure 1H).

Following tail vein injection of DiR-labeled ECs-derived Exos into CKD mice, we observed their accumulation in the aorta (Figure 1I), where they were subsequently internalized by VSMCs (Figure 1J).

### HP-exos derived from ECs drive VC in CKD mice

**FIGURE 2:**
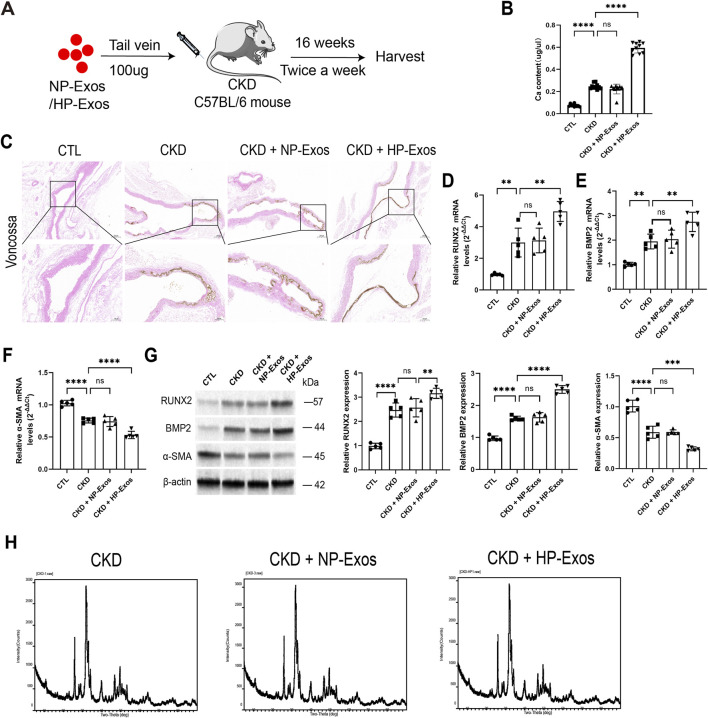
HP-Exos derived from ECs Drive VC in CKD mice. **(A)** Scheme of Exos injection in mice (n = 10 per group). **(B)** The content of calcium ion in the main artery of mice was measured. HP-Exos increased Ca content in aorta. **(C)** Voncossa staining (scale bar = 100 μm and 200 μm). HP-Exos increased mineralized nodules in aorta **(D-F)** RUNX2, BMP2 and α-SMA mRNA were indicated by qPCR. HP-Exos upregulated RUNX2 and BMP2, while downregulated α-SMA. **(G)** RUNX2, BMP2 and α-SMA protein were indicated by Western blot. HP-Exos upregulated RUNX2 and BMP2, while downregulated α-SMA. **(H)** X-ray diffraction analysis found that the composition of the calcified material was hydroxyapatite. Data represented mean ± SD. **p < 0.01, ***p < 0.001,****p < 0.0001, one-way ANOVA.

To investigate the role of ECs-derived Exos in VC, mice received twice-weekly tail vein injections of either NP-Exos or HP-Exos (Figure 2A). Body weight (measured on day 7 of each week.) in CTL group gradually increased, while decreased in CKD group. However, there were no significant differences among CKD groups receiving different Exos treatments (Supplementary Figure S1A). HP-Exos significantly increased aortic calcium content (Figure 2B) and enhanced mineralized nodule formation (Figure 2C). At the molecular level, HP-Exos upregulated RUNX2 and BMP2 expression (mRNA and protein levels) while downregulating α-SMA (Figures 2D–G). In contrast, NP-Exos showed no pro-calcific effects. X-ray diffraction confirmed hydroxyapatite as the predominant crystalline phase (Figure 2H). These findings demonstrate that HP-Exos derived from ECs potently exacerbate VC in CKD mice, with hydroxyapatite serving as the primary mineral component.

### HP-exos derived from ECs promote osteogenic differentiation of VSMCs

**FIGURE 3:**
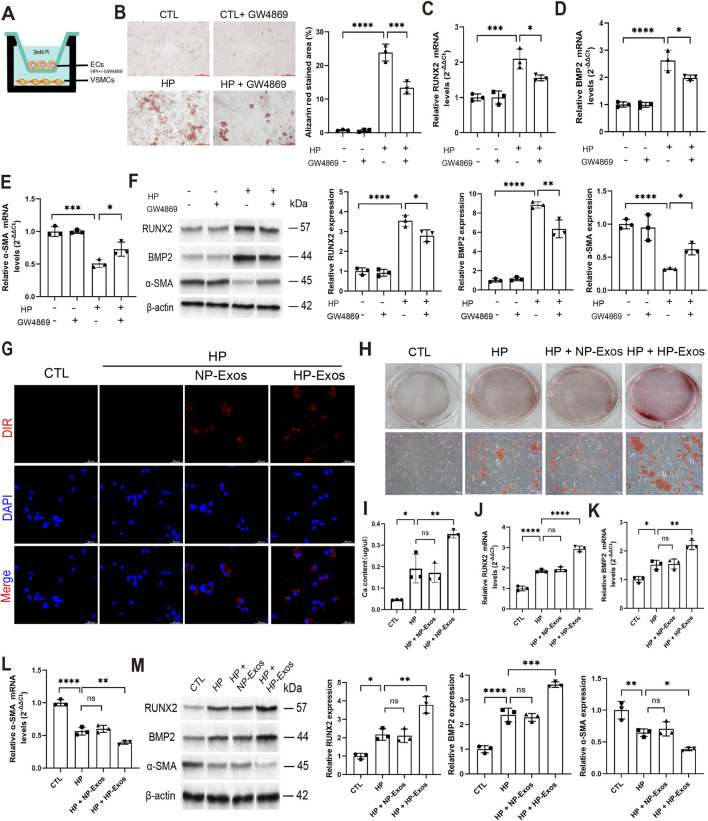
HP-Exos derived from ECs promote osteogenic differentiation of VSMCs*.* **(A)** Model of co-culture of ECs and VSMCs treated with or without Exos blocker GW4869 in a six-well transwell unit (n = 3 per group). **(B)** Alizarin Red S staining (scale bar = 100 μm). HP increased mineralized nodules in VSMCs. GW4869 mitigated the HP-induced effects. **(C-E)** RUNX2, BMP2 and α-SMA mRNA were indicated by qPCR. HP increased RUNX2 and BMP2, decreased α-SMA in VSMCs. GW4869 mitigated the HP-induced effects. **(F)** RUNX2, BMP2 and α-SMA protein were indicated by Western blot. HP increased RUNX2 and BMP2, decreased α-SMA in VSMCs. GW4869 mitigated the HP-induced effects. **(G)** VSMCs were incubated with Exos for 12 h (DIR in red, DAPI in blue, n = 3 per group, scale bar = 50 μm). **(H)** Alizarin Red S staining (scale bar = 100 μm, white arrow: calcification nodules). **(I)** The calcium content was determined. HP-Exos increased Ca content in VSMCs. **(J-L)** RUNX2, BMP2 and α-SMA mRNA were indicated by qPCR.HP-Exos upregulated RUNX2 and BMP2, while downregulated α-SMA. **(M)** RUNX2, BMP2 and α-SMA protein were indicated by Western blot. HP-Exos upregulated RUNX2 and BMP2, while downregulated α-SMA. Data represented mean ± SD. *p < 0.05, **p < 0.01, ***p < 0.001, ****p < 0.0001, one-way ANOVA.

To examine the crosstalk between ECs and VSMCs under HP conditions, we established a transwell co-culture system (Figure 3A). HP stimulation significantly increased mineralized nodule formation (Figure 3B), an effect that was attenuated by GW4869 (an secretion inhibitor of Exos). Molecular analysis revealed that HP treatment upregulated both mRNA and protein expression of osteogenic markers (RUNX2 and BMP2) while downregulating the VSMCs contractile marker α-SMA (Figures 3C–F).

To further investigate the direct effects of ECs derived Exos on VSMCs calcification, we treated VSMCs with isolated Exos. To confirm Exos uptaken by VSMCs, Exos were labeled with DIR and revealed their efficient internalization by VSMCs (Figure 3G). Our results demonstrated that HP-Exos significantly enhanced mineralization (Figure 3H) and increased calcium deposition On 7th day (Figure 3I). Meanwhile, HP-Exos markedly upregulated both mRNA and protein expression of osteogenic markers RUNX2 and BMP2 (Figures 3J–L) and downregulated the contractile marker α-SMA at both transcriptional and translational levels (Figures 3J–M). Notably, NP-Exos showed no significant effects on these calcification parameters. These *in vitro* findings provide direct evidence that Exos derived from HP-stimulated ECs promote VC *in vitro*.

### Exosomal miR-299-3p derived from ECs mediates VC

**FIGURE 4:**
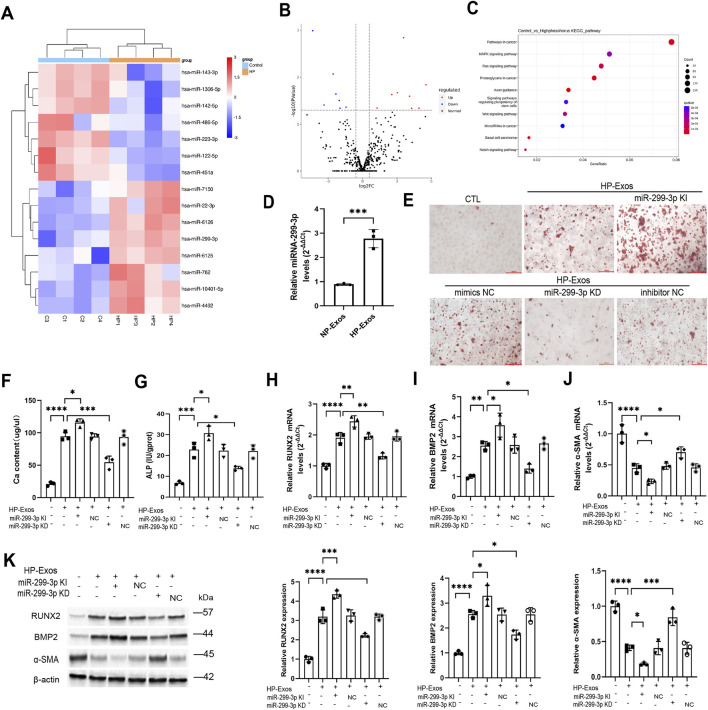
Exosomal miR-299-3p derived from ECs mediates VC. **(A)** The miRNA expression profile was sequenced to investigate the differentially expressed miRNAs, showed in Heat map (n = 4 per group). **(B)** Volcano map. **(C)** KEEG analysis. **(D)** MiR-299-3p level in Exos (n = 3 per group). miRNA-299-3p was upregulated or downregulated in ECs by miR-299-3p mimics or inhibitors. We exposed VSMCs to Exos isolated from ECs. Then, we exploded the caicification of VSMCs. **(E)** Alizarin Red S staining (scale bar = 100 μm). **(F)** Calcium content was determined (n = 3 per group). Upregulating miR-299-3p increased Ca content. Downregulating miR-299-3p had the inverse effects. **(G)** ALP activity was determined. Upregulating miR-299-3p increased ALP activity. Downregulating miR-299-3p had the inverse effects. **(H-J)** RUNX2, BMP2 and α-SMA mRNA were indicated by qPCR. Upregulating miR-299-3p increased RUNX2 and BMP2, while decreased α-SMA. Downregulating miR-299-3p had the inverse effects. **(K)** RUNX2, BMP2 and α-SMA protein were indicated by Western blot. Upregulating miR-299-3p increased RUNX2 and BMP2, while decreased α-SMA. Downregulating miR-299-3p had the inverse effects. CTL: control group (VSMCs treated with Exos from untransfected ECs); miR-299-3p Kl: VSMCs treated with Exos from ECs transfected with miR-299-3p mimics (knock-in/overexpression); mimics NC: VSMCs treated with Exos from ECs transfected with negative control mimics; miR-299-3p KD: VSMCs treated with Exos from ECs transfected with miR-299-3p inhibitor (knockdown); inhibitor NC: VSMCs treated with Exos from ECs transfected with negative control inhibitor. Data represented mean ± SD. *p < 0.05, **p < 0.01, ***p < 0.001, ****p < 0.0001, one-way ANOVA.

Exos regulate VC through intercellular communication, with miRNAs potentially serving as key mediators. miRNA sequencing of NP-Exos and HP-Exos identified eight significantly upregulated and seven downregulated miRNAs in HP-Exos (Figures 4A,B). These miRNAs were enriched in calcification-related pathways (MAPK, Wnt, Notch) (Figure 4C; Supplementary Figure S2), including miR-299-3p which showed prominent expression (Figure 4D; Supplementary Figure S3). To further investigate the mechanistic role of ECs-derived exosomal miR-299-3p in VC, we first performed transfection experiments to overexpress and knock down miR-299-3p in ECs, respectively (Supplementary Figure S4). Functional studies demonstrated that miR-299-3p overexpression in ECs-derived Exos promoted VC, as evidenced by increased mineralized nodules (Figure 4E), calcium deposition (Figure 4F), ALP activity (on 7th day) (Figure 4G), and osteogenic markers (RUNX2/BMP2) (Figures 4H,I,K), while suppressing α-SMA (Figures 4J,K). Conversely, miR-299-3p knockdown attenuated these pro-calcific effects, establishing ECs-derived exosomal miR-299-3p as a critical regulator of VC.

### MiR-299-3p mediates VC via targeting MARCH3

**FIGURE 5:**
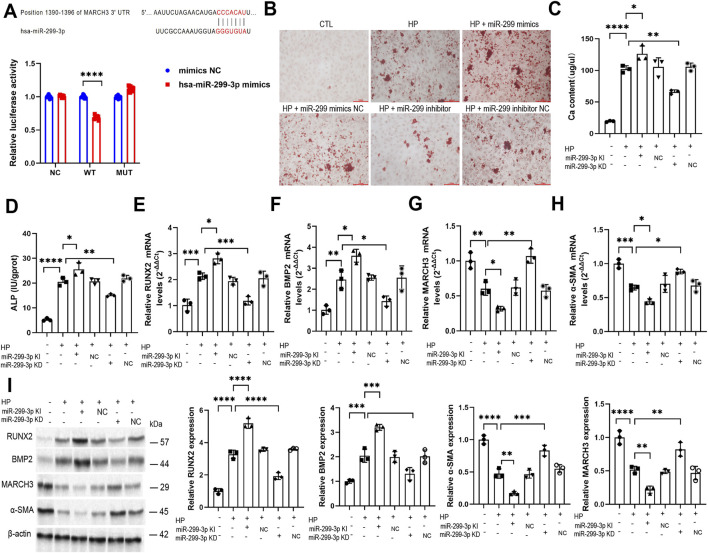
MiR-299-3p mediates VC via targeting MARCH3. **(A)** Targetscan predicted downstream target genes of miR-299-3p. Dual luciferase gene reporting experiment verified the targeting of miR-299-3p and MARCH3. **(B)** Alizarin Red S staining (scale bar = 100 μm). **(C)** Calcium content was determined (n = 3 per group). Upregulating miR-299-3p Ca contnet. Downregulating miR-299-3p had the inverse effects. **(D)** ALP activity was determined. Upregulating miR-299-3p increased ALP activity. Downregulating miR-299-3p had the inverse effects. **(E-H)** RUNX2, BMP2, MARCH3 and α-SMA mRNA were indicated by qPCR. Upregulating miR-299-3p increased RUNX2 and BMP2, while decreased α-SMA and MARCH3. Downregulating miR-299-3p had the inverse effects. **(I)** RUNX2, BMP2, MARCH3 and α-SMA protein were indicated by Western blot. Upregulating miR-299-3p increased RUNX2 and BMP2, while decreased α-SMA and MARCH3. Downregulating miR-299-3p had the inverse effects. Data represented mean ± SD. *p < 0.05, **p < 0.01, ***p < 0.001, ****p < 0.0001, one-way ANOVA.

Bioinformatic analysis using TargetScan predicted MARCH3 as a downstream target of miR-299-3p, which was experimentally validated through dual-luciferase reporter assays (Figure 5A). Subsequent functional studies in VSMCs with modulated miR-299-3p expression (Supplementary Figure S5) demonstrated its critical regulatory role. Overexpression of miR-299-3p significantly enhanced calcification parameters, including increased Alizarin red S-stained mineralized nodules (Figure 5B), elevated calcium content (Figure 5C), and heightened ALP activity (Figure 5D), while concurrently upregulating osteogenic markers RUNX2 and BMP2 (Figures 5E,F,I) and suppressing both α-SMA and MARCH3 expression (Figures 5G–I). Conversely, miR-299-3p inhibition produced the opposite effects, reducing calcification metrics while restoring α-SMA and MARCH3 levels. These complementary gain and loss of function experiments conclusively establish that miR-299-3p regulates VC through direct targeting of MARCH3 in VSMCs.

### MiR-299-3p contributes to VC via targeting MARCH3 *in vivo*

**FIGURE 6:**
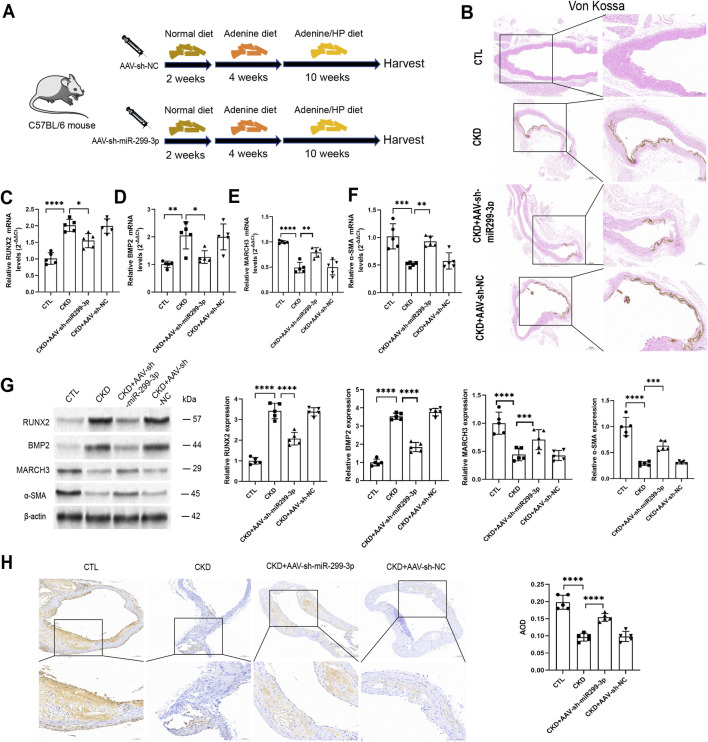
MiR-299-3p contributes to VC via targeting MARCH3 *in vivo*. **(A)** miR-299-3p AAV mouse model (n = 5 per group). **(B)** Voncossa staining (scale bar = 100 and 200 μm). **(C-F)** mRNA of RUNX2, BMP2, MARCH3 and α-SMA were indicated by qPCR (n = 5 per group). Downregulating miR-299-3p decreased RUNX2 and BMP2, while increased α-SMA and MARCH3. **(G)** RUNX2, BMP2, MARCH3 and α-SMA protein were indicated by Western blot. Downregulating miR-299-3p decreased RUNX2 and BMP2, while increased α-SMA and MARCH3. **(H)** IHC of MARCH3 (scale bar = 100 and 50 μm). Data represented mean ± SD. *p < 0.05, **p < 0.01, ***p < 0.001, ****p < 0.0001, one-way ANOVA.

To investigate the role of miR-299-3p in VC under pathological conditions, we established an *in vivo* model using AAV-mediated miR-299-3p knockdown of VSMCs (Figure 6A; Supplementary Figure S6). After miR-299-3p knockdown, the decrease in body weight was partially attenuated (Supplementary Figure S1B). This intervention resulted in significant attenuation of VC, as evidenced by markedly reduced calcium salt deposition (Figure 6B). Molecular analyses demonstrated that miR-299-3p knockdown consistently upregulated both mRNA and protein expression of MARCH3 and the contractile marker α-SMA, while significantly downregulating the osteogenic markers RUNX2 and BMP2 (Figures 6C–G). IHC analysis further confirmed these findings, showing decreased MARCH3 expression in CKD mice that was substantially restored following miR-299-3p inhibition (Figure 6H). These *in vivo* results corroborate our *in vitro* observations and collectively demonstrate that miR-299-3p promotes VC through targeted suppression of MARCH3.

### MARCH3 promotes transdifferentiation of VSMCs through p-JAK2/STAT5 pathway

**FIGURE 7:**
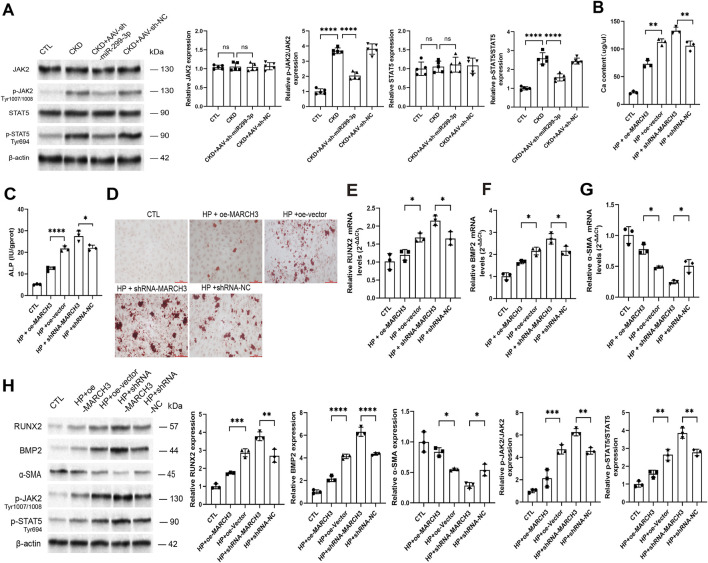
MARCH3 promotes transdifferentiation of VSMCs through p-JAK2/STAT5 pathway. **(A)** The expression level of proteins (n = 3 per group). Downregulating miR-299-3p decreased p-JAK2 and p-STAT5. **(B)** The Ca content was determined. Upregulating MARCH3 decreased Ca content. Downregulating miR-299-3p had the inverse effects. **(C)** The ALP activity was determined. Upregulating MARCH3 decreased ALP activity. Downregulating miR-299-3p had the inverse effects. **(D)** Alizarin red S staining (scale bar = 100 μm). **(E-G)** RUNX2, BMP2 and α-SMA mRNA were indicated by qPCR. Upregulating MARCH3 decreased RUNX2 and BMP2, while increased α-SMA. Downregulating miR-299-3p had the inverse effects. **(H)** RUNX2, BMP2, α-SMA, p-JAK2 and p-STAT5 were indicated by Western blot. Upregulating MARCH3 decreased RUNX2, BMP2, p-JAK2 and p-STAT5, while increased α-SMA. Downregulating miR-299-3p had the inverse effects. Data represented mean ± SD. *p < 0.05, **p < 0.01, ***p < 0.001, ****p < 0.0001, one-way ANOVA.

The JAK/STAT signaling pathway has been established as a critical mediator in chronic inflammatory and metabolic diseases including diabetes, atherosclerosis, and VC (Vigili de Kreutzenberg et al., 2022). Our investigation of this pathway following miRNA-299-3p downregulation in mice revealed a specific activation pattern: while total protein expression remained unchanged, we observed significant phosphorylation of receptor-associated Janus kinase (JAK) 2 at Tyr1007/1008 and signal transducers and activators of transcription (STAT) 5 at Tyr694 (Figure 7A). These findings prompted us to examine the functional relationship between MARCH3 and JAK/STAT signaling in VSMCs.

Through targeted modulation of MARCH3 expression in VSMCs (Supplementary Figure S7), we demonstrated that downregulation of MARCH3 significantly enhanced calcification parameters, including increased calcium content (Figure 7B), elevated ALP activity (Figure 7C), and augmented mineralized nodule formation (Figure 7D). At the molecular level, downregulation of MARCH3 upregulated RUNX2 and BMP2 mRNA while downregulating α-SMA expression (Figures 7E–G), and correspondingly increased protein levels of RUNX2, BMP2, p-JAK2, and p-STAT5 (Figure 7H) without affecting their non-phosphorylated forms (Supplementary Figure S8). Conversely, upregulation of MARCH3 produced the opposite effects, suppressing p-JAK2/STAT5 activation and mitigating VC progression. These results collectively establish that downregulated MARCH3, functioning downstream of miR-299-3p, promotes VC through specific activation of the p-JAK2/STAT5 signaling pathway. The selective phosphorylation of JAK2 and STAT5 without changes in their total protein levels suggests that MARCH3 mediates its effects through post-translational modification rather than transcriptional regulation of these signaling components.

## Discussion

Vascular calcification, as a life-threatening complication of CKD, markedly elevates cardiovascular morbidity and mortality in affected patients (Moncla et al., 2023; Carney, 2020; Kalantar-Zadeh et al., 2021). This study elucidates a novel mechanism by which ECs-derived Exos mediate CKD-VC under HP conditions. We demonstrate that ECs derived Exos facilitate VC progression via miR-299-3p, which directly targets MARCH3 to activate the p-JAK2/STAT5 signaling axis. Our findings establish the miR-299-3p/MARCH3/p-JAK2/STAT5 pathway as a critical regulatory mechanism in CKD-VC. Crucially, this work highlights the previously unrecognized role of exosomal crosstalk between ECs and VSMCs in driving VC pathogenesis, while identifying miR-299-3p as a key mediator of this intercellular communication. These findings provided a new mechanistic understanding of VC and might propose novel therapeutic avenues for CKD-VC.

While previous research emphasized osteogenic transdifferentiation of VSMCs, emerging evidence underscores ECs as critical regulators of VC initiation (Feenstra et al., 2024). Positioned at the vascular interface, ECs are primary sensors of hemodynamic and metabolic disturbances in CKD, including HP-induced stress. ECs regulate VC through multifaceted pathways involving inflammatory activation, oxidative stress, and dysregulated Wnt/β-catenin and BMP/Smad signaling (Faleeva et al., 2024; Chen et al., 2023). Our work extends this paradigm by demonstrating that ECs-derived Exos exhibit aortic tropism in murine models, directly promoting osteoblastic differentiation of VSMCs and VC.

Exos, enriched with miRNAs and bioactive cargos, are pivotal mediators of intercellular signaling in vascular pathology (Searles et al., 2024; Xu et al., 2024; Lu et al., 2024). Recent studies implicate exosomal transfer of osteogenic proteins (e.g., BMP2), miRNAs, and inflammatory mediators (e.g., IL-6) in VSMCs phenotypic modulation and VC progression (Koide et al., 2023; Buffolo et al., 2022). For instance, ECs-derived exosomal miR-204-5p mitigates atherosclerosis by suppressing VSMC calcification (Tian et al., 2024). Based on these observations, we proposed that HP stress might trigger ECs to release Exos with potent calcific potential in CKD. Using advanced Exos-tracking methodologies, we confirmed VSMCs uptake of ECs-derived Exos and revealed their cardiovascular targeting specificity *in vivo*. Importantly, our data provide direct evidence of HP-induced arterial exosomal secretion driving VC development.

Exosomal miRNA profiling identified miR-299-3p as markedly upregulated under HP conditions. Functional studies confirmed that miR-299-3p suppression attenuates CKD-VC progression. While tumor-suppressive roles of miR-299-3p documented (Wang et al., 2024; Wang B. et al., 2022; Tang et al., 2022), its vascular functions remain underexplored. Zhu et al. recently linked miR-299-3p to impaired efferocytosis in atherosclerosis via CD47 regulation (Zhu et al., 2024). Our present work establishes a novel VC-promoting role for ECs-derived exosomal miR-299-3p, revealing its capacity to drive medial calcification through VSMCs targeting. This discovery underscores the cooperative interplay between vascular cell populations within the calcific microenvironment, expanding our understanding of multicellular pathogenesis of VC.

Mechanistically, miR-299-3p directly targets MARCH3, a member of the E3 ubiquitin ligase family implicated in immune regulation and membrane trafficking (Zeng et al., 2022; Feng et al., 2022). Members of the MARCH family are involved in diverse cellular functions, such as immune regulation, protein quality control, and membrane transport (Lin et al., 2018). Previous studies have demonstrated the role of MARCH3 in some processes, including endoplasmic reticulum-related degradation, endosomal protein transport, the balance of mitochondrial dynamics, and spermatogenesis (Lin et al., 2021). However, the potential involvement of MARCH3 in CKD-VC remained unexplored. We demonstrate that MARCH3 depletion exacerbates VC, whereas its overexpression attenuates calcification.

Research has found that deletion of MARCH3 negatively regulates the degradation of IL-3α and stabilizes IL-3α via reduced ubiquitination. Our data also showed that IL-3Rα protein levels were downregulated upon MARCH3 overexpression. Conversely, IL-3Rα protein levels were upregulated upon MARCH3 knockdown (Supplementary Figure S9). Downregulation of MARCH3 also activates JAK family members in a sepsis model of cecal ligation and puncture, which leads to the phosphorylation of STAT (Tobin and Cooper, 2024). In addition, it was found that STAT1 can drive the osteoblast-transdifferentiation of VSMCs. Moreover, activation of JAK2/STAT3 has been shown to accelerate VC (Zuo et al., 2025; Hao et al., 2023). Upon phosphorylation, JAK/STAT proteins dimerize and directly translocate into the nucleus, where they bind to specific STAT response elements within the RUNX2 gene promoter, thereby transcriptionally activating RUNX2 expression. As the core transcription factor of osteogenic differentiation, RUNX2 subsequently initiates the transcriptional cascade of downstream osteogenic markers, such as BMP2. Furthermore, STAT proteins can also cooperate to enhance the expression of osteogenic genes by inducing auxiliary transcription factors, including C/EBPδ and C/EBPβ (Vigili de Kreutzenberg et al., 2022). Our data reveal that downregulation of MARCH3 enhances phosphorylation of JAK2/STAT5. Importantly, this pathway was activated in CKD-VC models and suppressed following MARCH3 inhibition, highlighting its functional significance in the disease process. The findings position p-JAK2/STAT5 signaling as a critical downstream effector of the miR-299-3p/MARCH3 axis in VC.

We acknowledge certain limitations in the present study. The CKD mouse model employed, while well-established, induces kidney injury over a relatively compressed timeframe of 16 weeks, which may different with the typically chronic progression of human CKD. Consequently, although this model effectively recapitulates key features of CKD-VC, its nature may not fully capture the complex pathophysiology of the human disease. Future studies utilizing more slowly progressive models or clinical samples will be important to further validate the translational relevance of our findings.

## Conclusion

**FIGURE 8:**
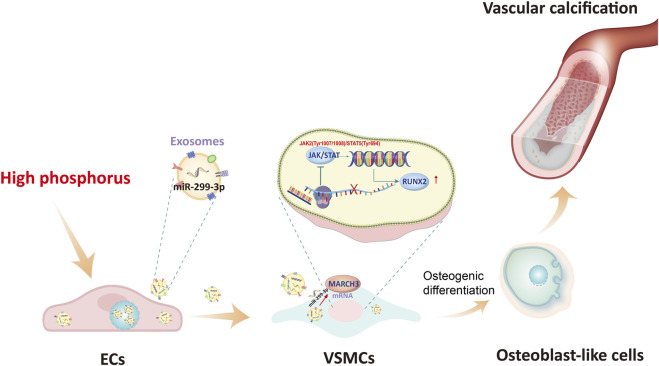
The proposed mechanism of VC mediated by crosstalk between ECs and VSMCs via exosomal miR-299-3p. HP-stimulated ECs release exosomes containing miR-299-3p; Exosomes are taken up by VSMCs; miR-299-3p targets and downregulates MARCH3; Reduced MARCH3 leads to activation of p-JAK2/p-STAT5 signaling; Activated STAT5 translocates to the nucleus; Increased transcription of osteogenic markers (RUNX2); VSMCs undergo osteoblast-like transdifferentiation; Vascular calcification progression.

In summary, we demonstrated a pathogenic cascade wherein HP-stimulated ECs release exosomal miR-299-3p, which is internalized by VSMCs to suppress MARCH3 expression. This inhibition activates p-JAK2/STAT5 signaling, driving osteogenic transdifferentiation of VSMCs and VC progression (Figure 8). Our findings not only unravel a novel Exos-mediated ECs-VSMCs crosstalk mechanism in CKD-VC but also identify exosomal miR-299-3p and MARCH3 as promising therapeutic targets. Preserving ECs homeostasis and targeting exosomal signaling pathways may offer innovative strategies for mitigating VC in CKD patients.

## Data Availability

The data presented in the study are deposited indeposited in the Genome Sequence Archive (GSA) at the National Genomics Data Center, China National Center for Bioinformation / Beijing Institute of Genomics, Chinese Academy of Sciences, under accession number CRA040855. The data are publicly accessible at https://ngdc.cncb.ac.cn/gsa. The shared URL is: https://ngdc.cncb.ac.cn/gsa/s/0XFF41PH. Any queries / additional information can be obtained from the corresponding author upon request.
