# Supplementary material for: Exosomal hsa-miR-299-3p from endothelium mediates high phosphorus-induced vascular calcification in mice model of CKD via phosphorylated JAK2/STAT5 pathway
Source: Front Pharmacol. 2026 Apr 9;17:1752954. doi: 10.3389/fphar.2026.1752954 (PMC13103977; doi:10.3389/fphar.2026.1752954)
Supplement: Supplementary file 1 [file Supplementaryfile1.pdf]

SUPPLEMENTAL FIGURES:

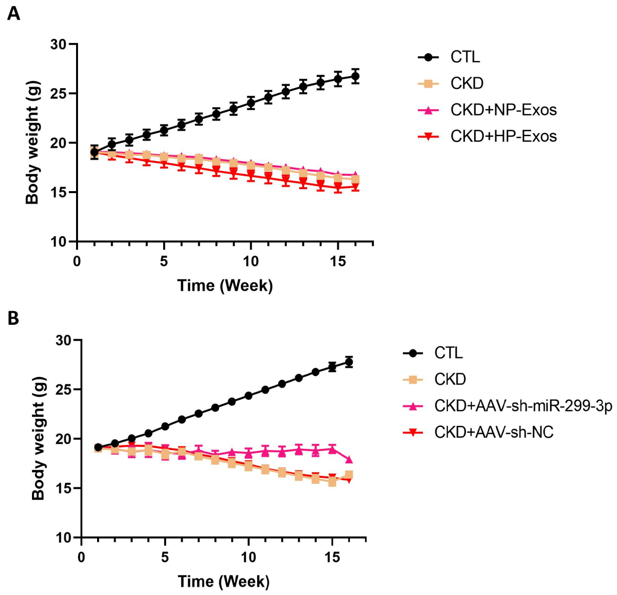

Figure S1. Mouse body weight monitoring. Body weight was measured on day 7 of each week throughout the 16-week experimental period. **A** CKD mice received twice-weekly tail vein injections (NP-Exos or HP-Exos). During this process, changes in mouse body weight were monitored and recorded. **B** We established an in vivo model using AAV-mediated miR-299-3p knockdown in vascular smooth muscle cells and monitored the body weight of mice in each group.

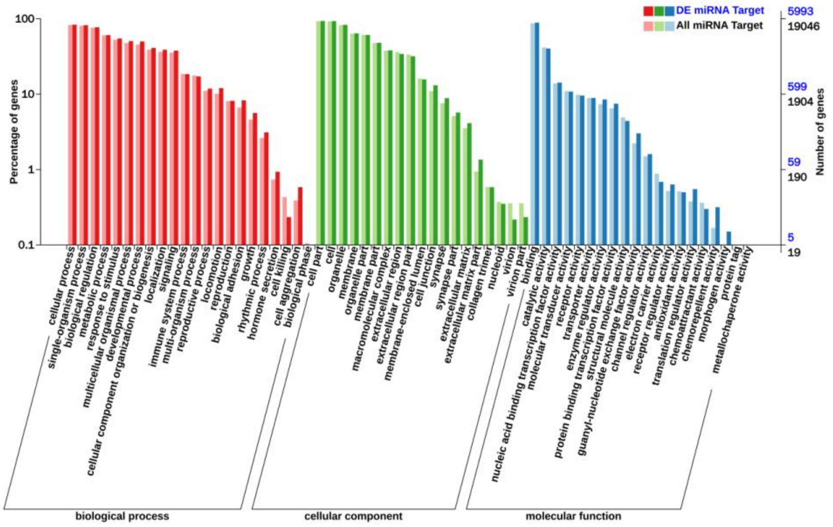

Figure S2. Analysis of miRNA sequencing on NP-Exos and HP-Exos. GO analysis of different miRNA in NP-Exos and HP-Exos.

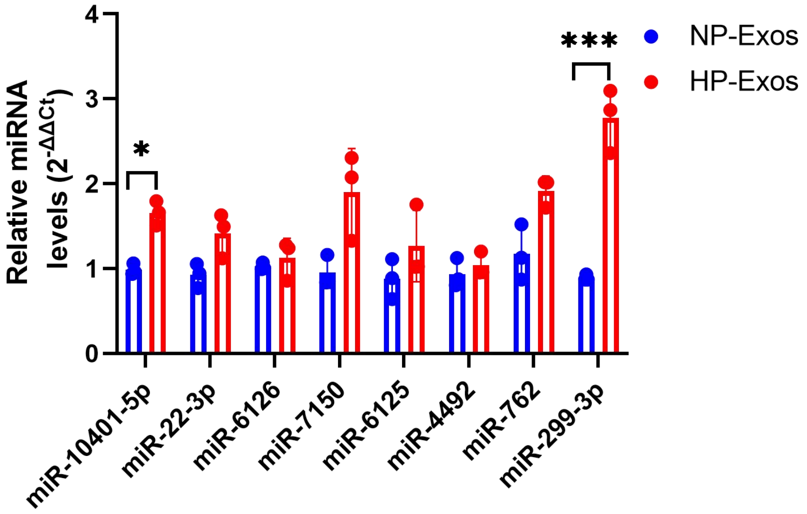

Figure S3. Expression level of miR-10401-5p, miR-22-3p, miR-6126, miR-7150, miR-6125, miR-4492, miR-762 and miR-299-3p in NP-EC-Exos and HP-ECs-Exos. MRNA levels were analysed by qRT-PCR. Data are shown as mean ± SD, with three independent experiments. *p < 0.05, ***p < 0.001, unpaired two-tailed Student's t-test.

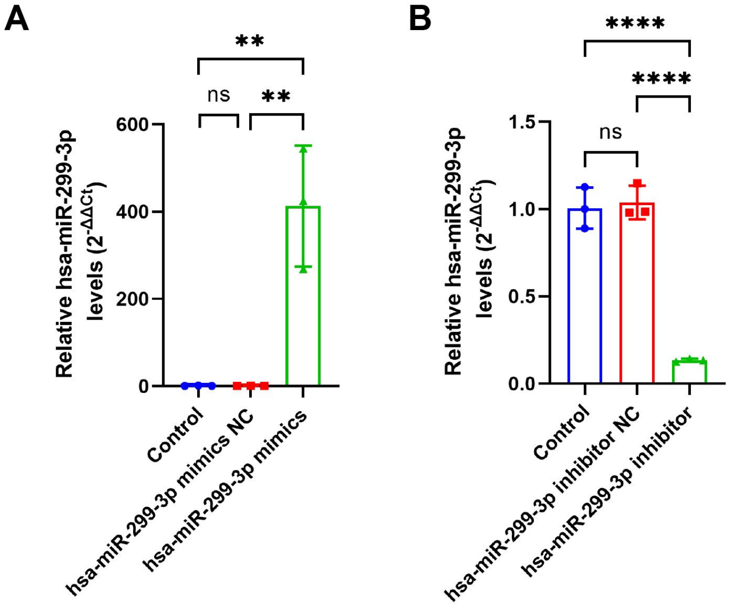

Figure S4. miR-299-3p transfection in ECs. Transfection efficiency was assessed after 48 hours. **A** miR-299-3p mimics transfection in ECs (n=3 per group). **B** miR-299-3p inhibitor transfection in ECs (n=3 per group). Data are presented as mean±SD. **p < 0.01, ****p < 0.0001, ns: no significance, one-way ANOVA.

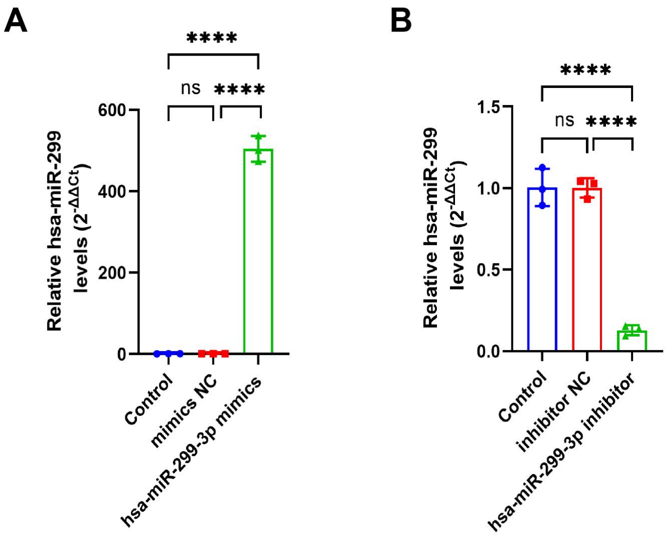

Figure S5. miR-299-3p transfection in VSMCs. Transfection efficiency was assessed after 48 hours. **A** miR-299-3p mimics transfection in VSMCs (n=3 per group). **B** miR-299-3p inhibitor transfection in VSMCs (n=3 per group). Data are presented as mean±SD. ****p < 0.0001, ns: no significance, one-way ANOVA.

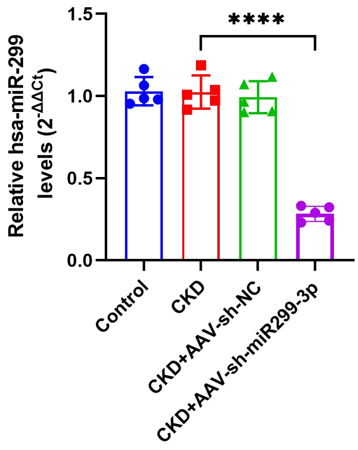

Figure S6. miR-299-3p knockdown mice. We generated miR-299-3p down-regulated mice with AAV and randomly divided the animals into several groups with different treatments (n = 5 per group). Transfection efficiency was assessed before modeling. Data are presented as mean±SD. ****p < 0.0001, one-way ANOVA.

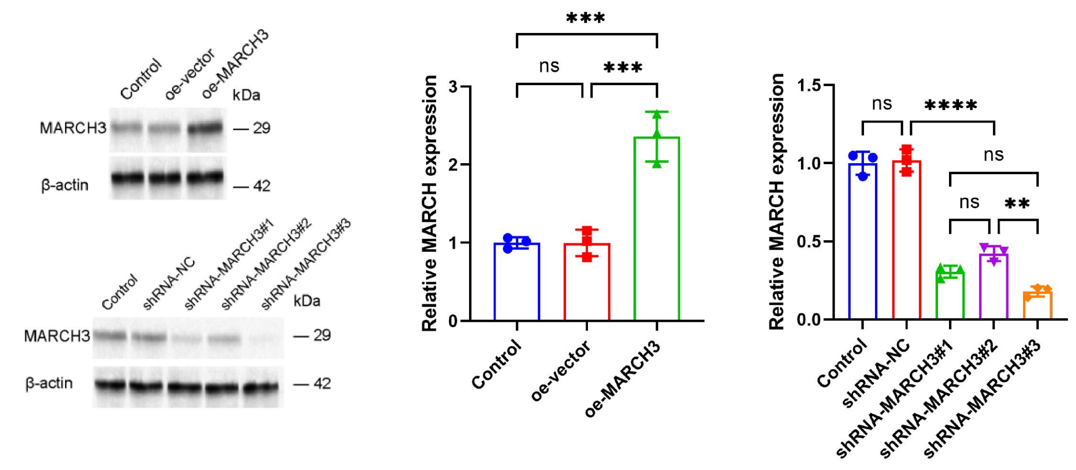

Figure S7. MARCH3 transfection validation in VSMCs (n=3 per group). Transfection efficiency was assessed after 48 hours. The expression level of MARCH3 were indicated by western blot. Data are presented as mean±SD. $^{**}p < 0.01$, $^{***}p < 0.001$, $^{****}p < 0.0001$, ns: no significance, one-way ANOVA.

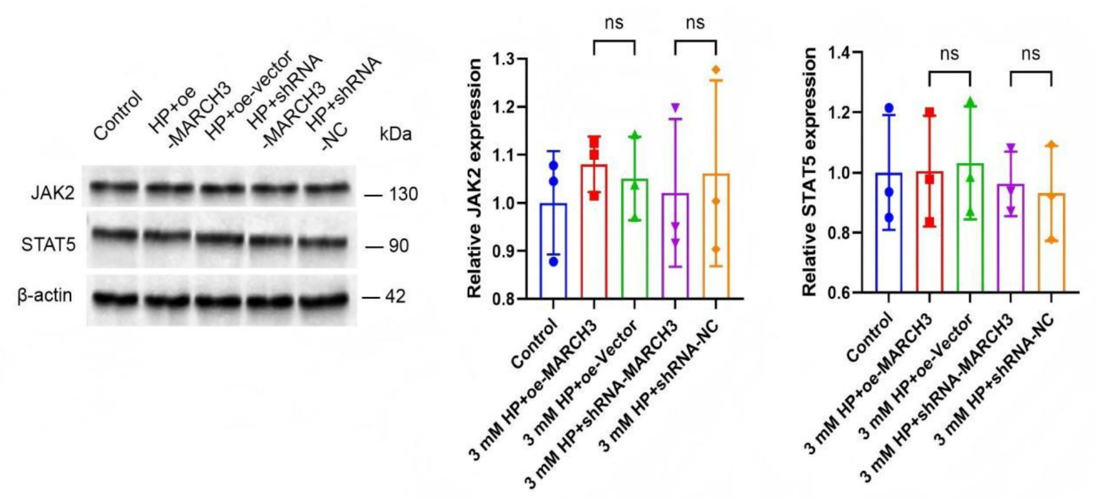

Figure S8. MARCH3 regulated JAK2/STAT5 pathway. The expression level of JAK2 and STAT5 were indicated by western blot. Data are presented as mean±SD. Ns: no significance, one-way ANOVA.

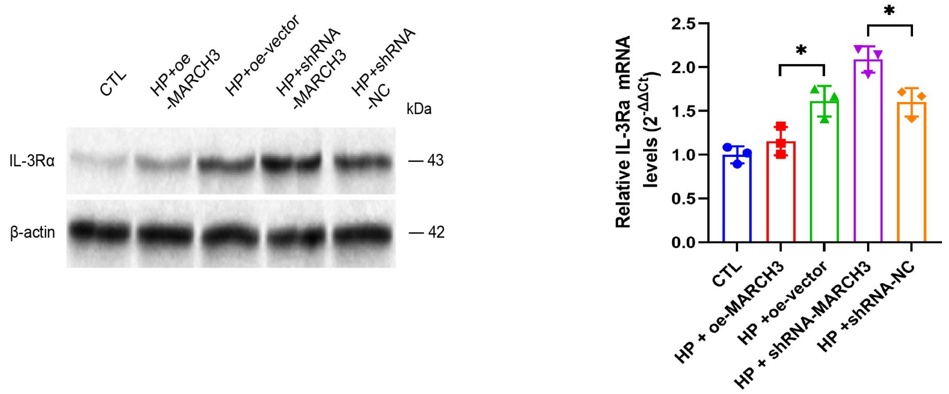

Figure S9. MARCH3 regulated IL3Rα. The expression level of IL3Rα was indicated by western blot. Data are presented as mean±SD. *p < 0.05, one-way ANOVA. (Figure S9 and Figure 7H originate from the same Western blot membrane and therefore share the same loading control bands.)

Supplemental method

Table S1. Primers used in Quantitative Real-Time PCR

Primers		Sequence (5'→3')
RUNX2 (human)	Forward	TCGCCTCACAAACAACCACA
	Reverse	CTGGTAGTGACCTGCGGAGA
BMP2 (human)	Forward	CTAAGGAGGACGACAGCACC
	Reverse	AAGAAGTCCCCAGCCAAGTG
α-SMA (human)	Forward	AAAGCAAGTCCTCCAGCGTT
	Reverse	TTCACAGGATTCTGGGAGCG
MARCH3 (human)	Forward	CGTTGAGCAGGTCCGTGAA
	Reverse	TCCCGATAGCAAACCGACG
GAPDH (human)	Forward	CCACATCGCTCAGACACCAT
	Reverse	AGTTAAAAGCAGCCCTGGTGA
RUNX2 (mouse)	Forward	ATGCTTCATTCGCCTCACAAACAA
	Reverse	TGCTTGCAGCCTTAAATATTCCTG
BMP2 (mouse)	Forward	AAACACAAACAGCGGAAGCG
	Reverse	CGATCCAGTCATTCCACCCC
α-SMA (mouse)	Forward	CGGGAGCAGAACAGAGGAAT
	Reverse	ACTCGCTTCCCAAACAAGGA
MARCH3 (mouse)	Forward	CGTGGATCACCTGCACTTTAG
	Reverse	ATCACCCTCTGATTGGTCCGA
GAPDH (mouse)	Forward	CCCTTAAGAGGGATGCTGCC
	Reverse	TACGGCCAAATCCGTTCACA

Table S2. Primers of miR-299-3p

Primers		Sequence (5'→3')
miR-299-3p	Forward	CGCGTATGTGGGACGGTAAA
	Reverse	AGTGCAGGGTCCGAGGTATT
U6	Forward	CTCGCTTCGGCAGCACA
	Reverse	AACGCTTCACGAATTTGCGT

Table S3. Antibodies

Antibodies	Cat. No.	Dilution (application)
TSG101	Abcam, ab125011	1:1000 (WB)
Alix	Abcam, ab186429	1:1000 (WB)
CD9	Abcam, ab263019	1:1000 (WB)
Calnexin	Proteintech, 10427-2-AP	1:1000 (WB)
RUNX2	Abcam, ab236639	1:1000 (WB)
BMP2	Abcam, ab284387	1:1000 (WB)
α-SMA	Affinity Biosciences, AF1032	1:500 (WB)
MARCH3	Affinity Biosciences, DF4019	1:500 (WB)
JAK2	Abcam, ab108596	1:5000 (WB)
Phospho-Jak2 (Tyr1007/1008)	Abcam, ab32101	1:1000 (WB)
STAT5	Affinity Biosciences, AF6305	1:500 (WB)
Phospho-Stat5 (Tyr694)	Affinity Biosciences, AF3305	1:500 (WB)
IL-3Rα	Abcam, ab200687	1:500 (WB)
β-actin	Abcam, ab8227	1:1000 (WB)
Goat Anti-Rabbit IgG H&L (HRP)	Abcam, ab6721	1:10000 (WB)
